# A new familial form of a late-onset, persistent hyperinsulinemic hypoglycemia of infancy caused by a novel mutation in *KCNJ11*

**DOI:** 10.1080/19336950.2017.1393131

**Published:** 2017-12-12

**Authors:** Yen-Yu Yang, Roger K. Long, Christine T. Ferrara, Stephen E. Gitelman, Michael S. German, Shi-Bing Yang

**Affiliations:** a Institute of Biomedical Sciences, Academia Sinica, Taipei, Taiwan; b Department of Pediatrics, University of California San Francisco, USA; c Diabetes Center, University of California San Francisco, USA; d Department of Medicine and Eli and Edythe Broad Center of Regeneration Medicine and Stem Cell Research, University of California San Francisco, USA

**Keywords:** ATP-Sensitive Potassium Channel (K_ATP_), Inwardly Rectifying Potassium Channel 6.2 (Kir6.2), Persistent Hyperinsulinemic Hypoglycemia of Infancy (PHHI), sulphonylurea Receptor 1 (SUR1)

## Abstract

The ATP-sensitive potassium channel (K_ATP_) functions as a metabo-electric transducer in regulating insulin secretion from pancreatic β-cells. The pancreatic K_ATP_ channel is composed of a pore-forming inwardly-rectifying potassium channel, Kir6.2, and a regulatory subunit, sulphonylurea receptor 1 (SUR1). Loss-of-function mutations in either subunit often lead to the development of persistent hyperinsulinemic hypoglycemia of infancy (PHHI). PHHI is a rare genetic disease and most patients present with immediate onset within the first few days after birth. In this study, we report an unusual form of PHHI, in which the index patient developed hyperinsulinemic hypoglycemia after 1 year of age. The patient failed to respond to routine medication for PHHI and underwent a complete pancreatectomy. Genotyping of the index patient and his immediate family members showed that the patient and other family members with hypoglycemic episodes carried a heterozygous novel mutation in *KCNJ11* (C83T), which encodes Kir6.2 (A28V). Electrophysiological and cell biological experiments revealed that A28V hKir6.2 is a dominant-negative, loss-of-function mutation and that K_ATP_ channels carrying this mutation failed to reach the cell surface. *De novo* protein structure prediction indicated that this A28V mutation reoriented the ER retention motif located at the C-terminal of the hKir6.2, and this result may explain the trafficking defect caused by this point mutation. Our study is the first report of a novel form of late-onset PHHI that is caused by a dominant mutation in *KCNJ11* and exhibits a defect in proper surface expression of Kir6.2.

## Introduction

Persistent hyperinsulinemic hypoglycemia of infancy, or PHHI, is the most common cause of severe neonatal hypoglycemia that lasts beyond the first a few hours of life.^1^ Both sporadic and familial forms of the disease exist, and several genes have been shown to be involved in the pathophysiology. Among these genes, disease-associated alleles for *KCNJ11, ABCC8, GLUD1*, and *GCK* are the most common factors that lead to the development of PHHI.^2,3^ Interestingly, the severity of this disease is highly variable, even among family members that carry the same mutated genes.^4^

*KCNJ11* and *ABCC8* genes encode the inwardly-rectifying potassium channel 6.2 (Kir6.2) and the sulphonylurea receptor 1 (SUR1), respectively, and co-assembly of four Kir6.2 with four SUR1 subunits forms a functional ATP-sensitive potassium channel (K_ATP_ channel).^5^ Intracellular ATP blocks the K_ATP_ channel via direct interaction with Kir6.2, the pore-forming subunit of the channel. While most excitable cells, including pancreatic β-cells, neurons, cardiac myocytes and skeletal muscles, have Kir6.2 as the K_ATP_ channel pore forming subunit, vascular smooth muscle cells have Kir6.1 instead.^6^ SUR1, on the other hand, is a regulatory protein that confers sensitivity to magnesium nucleotides and drugs, such as sulphonylureas and K_ATP_ channel openers.^7^ At the cellular level, the K_ATP_ channel functions as a metabo-electric transducer, since its gating is regulated by the intracellular metabolites such as ATP, long-chain fatty acid-CoA, and phosphatidylinositol-4,5-bisphosphate (PIP_2_).^8^ Given that K_ATP_ channels are involved in multiple physiological processes, it is not surprising that mutations in either *KCNJ11* or *ABCC8* can cause a variety of diseases, ranging from diabetes and PHHI to epilepsy, mental retardation (DEND syndrome) and cardiac myopathies.^9^

Metabolism of glucose by the pancreatic β-cells rapidly increase intracellular ATP ([ATP]_i_). Elevated [ATP]_i_ closes the K_ATP_ channels, which depolarizes the β-cell membrane potentials and subsequently opens the voltage-gated Ca^2+^ channels. The resulting influx of Ca^2+^ into the cytosol ultimately triggers insulin secretion via exocytosis.^10^ Because the K_ATP_ maintains the resting membrane potentials of the pancreatic β-cells, loss-of-function in either of the K_ATP_ channel subunits may lead to aberrant depolarization of β-cells and excessive insulin release.^3^ In contrast to mutations in *ABCC8*, most of the PHHI causing mutations in *KCNJ11* are recessive, requiring both parents to be carriers.^2,11^ In this study, we report a novel dominant form of PHHI, which is caused by a single point mutation (C83T) in *KCNJ11* that codes for a Val substitution for Ala at position 28 of the Kir6.2 peptide chain (A28V hKir6.2). The index patient, carrying a maternally inherited A28V hKir6.2, developed diazoxide-nonresponsive, late-onset PHHI requiring a total pancreatectomy. Immediate family members who carry the same heterozygous mutation have also experienced various degrees of hyperinsulinemic hypoglycemic symptomology. Moreover, both the patient and one of his affected siblings developed primary hypopituitarism. Electrophysiological and cell biological studies reveal that the A28V hKir6.2 mutation produces only minuscule K_ATP_ currents due to a trafficking defect that prevents surface expression. Our results identify a novel K_ATP_ channel defect that causes PHHI and provides additional evidence that the N-terminus of Kir6.2 is involved in K_ATP_ channel trafficking.

## Results

### Clinical findings

The male patient presented at 9 months old with failure-to-thrive, weight loss, and feeding intolerance. Initial evaluations revealed reflux esophagitis and a duodenal ulcer, but persistent symptoms eventually led to diagnoses of hyperinsulinemic hypoglycemia and central hypothyroidism at 16 months. The hypoglycemia was refractory to diazoxide therapy, and after three sequential partial pancreatectomies failed to control the hypoglycemia, removal of the last ∼1% of the pancreas successfully prevented further hypoglycemia and resulted in diabetes mellitus. Histologic examinations of the resected pancreas revealed islet cell hyperplasia. Post pancreatectomy, he developed intractable gastrointestinal bleeding from multiple sites along with hypergastrinemia eventually requiring resection of the stomach and intestine, which also showed endocrine cell hyperplasia.

Notable family history includes asymptomatic hypoglycemia in the mother which did not require treatment. An older sister was diagnosed at 12 months with central hypothyroidism and adrenal insufficiency but a normal pituitary on MRI scan. When she developed repeated severe hypoglycemia despite adequate thyroid, cortisol and growth hormone replacement, she was also diagnosed with hyperinsulinemic hypoglycemia, which also failed to respond to diazoxide.

**Figure 1.**
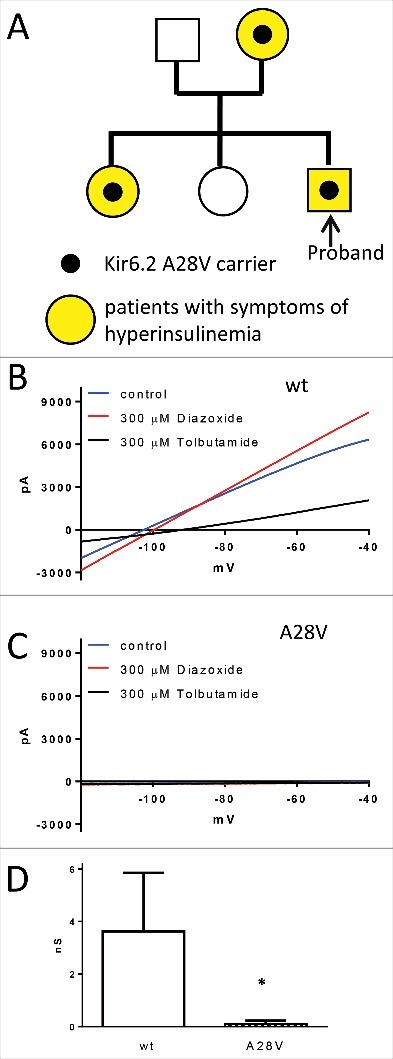
(A) The family pedigree of the index patient. Each family member that carries the A28V hKir6.2 mutation exhibits some degree of PHHI. (B to D) Whole-cell recording of K_ATP_ currents in HEK cells. (B) A representative wild-type K_ATP_ current (blue trace) was elicited by a voltage ramp pulse (0.5V/s). As predicted, the K_ATP_ current was augmented by 300 µM K_ATP_ channel opener diazoxide (red trace) and inhibited by 300 µM K_ATP_ channel blocker tolbutamide (black trace). (C) In contrast, HEK cells transfected with A28V hKir6.2 exhibited minuscule K_ATP_ current (blue trace) and neither 300 µM K_ATP_ channel opener diazoxide (red trace) or 300 µM K_ATP_ channel blocker tolbutamide (black trace) had an effect on the A28V K_ATP_ currents. (D) Summary of wild-type and A28V K_ATP_ currents in HEK cells. Wild-type K_ATP_ currents (n = 5) were significantly larger than A28V K_ATP_ currents (n = 9), as determined by Mann-Whitney U-test (*p < 0.05).

Commercial sequencing identified a heterozygous variant in *KCNJ11* (C83T) that encodes an Ala to Val substitution at amino acid 28 in the Kir6.2 protein in the proband. The patient's mother and sister carry the same A28V Kir6.2 mutation (Fig. 1A).

### Functional characterization of the hKir6.2 (A28V) mutation

Loss of K_ATP_ channel function is one of the most common causes of PHHI.^1^ We first expressed wild-type or mutant hKir6.2 with hSUR1 in HEK293 cells to examine whether the A28V mutation disrupts K_ATP_ channel function. In cells transfected with wild-type hKir6.2 and hSUR1, a weak-inwardly rectifying potassium current was observed, and this current was further augmented by diazoxide, a K_ATP_ channel opener, and inhibited by tolbutamide, a K_ATP_ channel blocker (Fig. 1B). In contrast, cells transfected with A28V hKir6.2 showed only minuscule potassium currents and adding diazoxide failed to augment the currents (Fig. 1C). These results indicated that A28V hKir6.2 does not form a functional K_ATP_ channel (Fig. 1D) in HEK cells.

### A28V hKir6.2 impairs K_ATP_ channel trafficking

**Figure 2.**
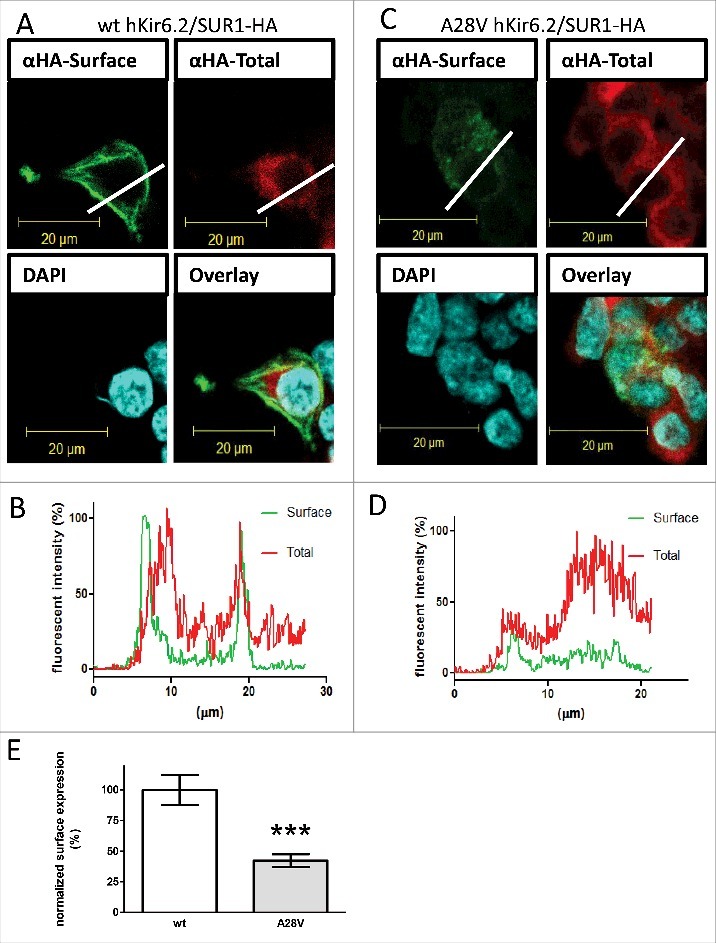
Surface expression of K_ATP_ channels in HEK cells. An HA-tag was inserted into the extracellular domain on rSUR1 and then co-transfected with either wild-type hKir6.2 (A) or A28V hKir6.2. As Kir6.2 must be co-assembled with SUR1 for correct trafficking, the subcellular distribution of HA staining should faithfully represent the K_ATP_ channel distribution. K_ATP_ channels located on the cell surface were labeled green. Total K_ATP_ channels were labeled red, and cell nuclei were counterstained with DAPI (blue). The wild-type K_ATP_ channels were clearly visible on the cell surface (A), but A28V hKir6.2-containing channels were not readily observed on the cell surface (C). (B&D): Cross-sectional staining intensity profiles of HEK cells expressing wild-type hKir6.2 (C) and A28V hKir6.2 (D). The profiles were determined from cross-sections indicated by the white lines in A and C. Surface staining signals are clearly visible in HEK cells transfected with wild-type hKir6.2, as the green line shows distinct peaks at the cell boundary. By contrast, in HEK cells transfected with A28V hKir6.2, the cell surface boundary is not clearly demarcated (green line, D). (E) Quantitative analysis of K_ATP_ channel surface staining signals. K_ATP_ channels containing A28V hKir6.2 had greatly reduced surface staining signals compared to wild-type hKir6.2 containing K_ATP_ channels (***p < 0.0005, Mann-Whitney U-test n = 11 for each group).

A lack of discernible K_ATP_ currents can result from defects in either channel conduction or channel trafficking.^3^ We next tested whether K_ATP_ channels containing A28V hKir6.2 would traffic to the cell membrane by staining K_ATP_ channels on the cell surface. To minimize artifacts that caused by manipulating the hKir6.2 subunit, we probed the subcellular location of the K_ATP_ channel using a HA-tagged rodent SUR1 (rSUR1) subunit. Prior to the trafficking from the endoplasmic reticulum (ER) to the plasma membrane via the Golgi complex, functional K_ATP_ channels must be co-assembled to mask strong ER-retention signals in Kir6.2 and SUR1 subunits.^12^ Therefore, tracking the HA-tagged SUR1 is a preferred method to assess the cellular localization of fully assembled K_ATP_ channels in the presence of Kir6.2 mutants. In the HEK cells transfected with wild-type hKir6.2 and HA-rSUR1, a very strong surface staining pattern was detected (Fig. 2A), as this surface signal clearly outlined the cell boundary (Fig. 2B). This result indicated that the K_ATP_ channels containing wild-type hKir6.2 can promote the forward trafficking of HA-tagged rSUR1 by forming functional K_ATP_ channels. However, for HEK293 cells transfected with A28V hKir6.2 and HA-rSUR1, most of the HA signal was intracellular (Fig. 2C and D). By normalizing the surface HA signal (green) to total HA signal (red), we found the proportion of HA signal at the cell surface was significantly reduced in cells with K_ATP_ channels containing A28V hKir6.2 (Fig. 2E). Based on these results, we conclude that A28V hKir6.2 impairs K_ATP_ channel function by suppressing surface expression in HEK cells.

### Dominant-negative effect of A28V hKir6.2 on K_ATP_ channel trafficking

**Figure 3.**
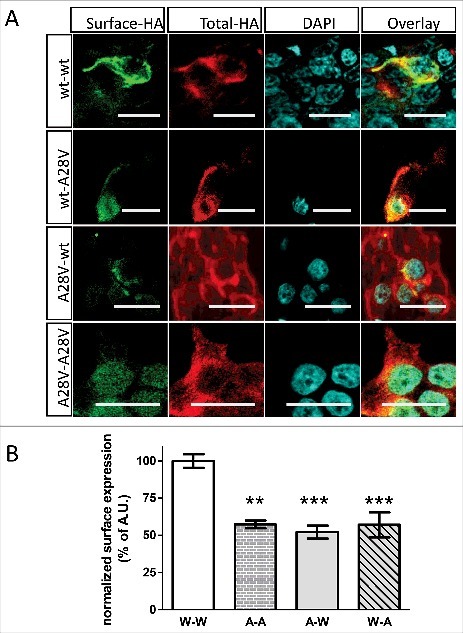
hKir6.2(A28V) has a dominant-negative effect on K_ATP_ channel surface expression in HEK cells. (A) An HA-tag was inserted into the extracellular domain of rSUR1 and then co-transfected with one of the hKir6.2 tandem constructs that are linked by a self-cleaving P2A linker. Only cells transfected with the wt-P2A-wt hKir6.2 construct showed strong surface staining (top row). Surface expression was not apparent in HEK cells expressing K_ATP_ channels that contain wt-P2A-A28V, A28V-P2A-wt or A28V-P2A-A28V hKir6.2. (B) Quantitative analysis of the surface signal from cells transfected with various hKir6.2 tandem constructs. K_ATP_ channels formed from wt-P2A-wt tandem construct expression had much stronger surface staining signals compared to any other hKir6.2 tandem constructs that contain the A28V Kir6.2 mutation. (**p = 0.001, ***p < 0.0005, one-way ANOVA with posthoc multiple comparison test, n = 8 for wt-P2A-wt, 8 for A28V-P2A-A28V, 9 for A28V-P2A-wt, and 14 for wt-P2A-A28V).

The patient and the other family members who exhibited hypoglycemia are all heterozygous for the *KCNJ11* (C83T) mutation, suggesting that A28V hKir6.2 is a dominant-negative mutation that can suppress normal K_ATP_ channel trafficking. To control the expression ratio of wild-type and hKir6.2 (A28V) in the HEK293 cells, we generated a construct to express wide-type and A28V hKir6.2 in tandem, with the two sequences linked by a self-cleaving P2A peptide linker. P2A encodes an RNA skipping signal, so during translation, the ribosome produces a gap in the nascent polypeptide. Thus the two peptide sequences that are linked by the P2A are “self-cleaved” at the P2A sequence and yield two physically separate peptides.^13^ We generated all four possible arrangements of wild-type and A28V hKir6.2 pairs and examined whether A28V hKir6.2 suppresses K_ATP_ channel trafficking to the plasma membrane. Our results showed that cells transfected with the wt-P2A-wt hKir6.2 tandem construct exhibited strong surface signals in HEK293 cells, while those transfected with the A28V-P2A-A28V hKir6.2 construct showed greatly reduced surface signals (Fig. 3A). These results further confirmed the effect of A28V-hKir6.2 in suppressing K_ATP_ channel trafficking (Fig. 2A and B). Interestingly, HEK293 cells transfected with either wt-P2A-A28V or A28V-P2A-wt constructs showed diminished surface signals (Fig. 3A), comparable to the HEK293 cells transfected with either A28V monomers (Fig. 2B) or A28V-P2A-A28V homo tandem construct (Fig. 3A and B). These results strongly suggested that A28V hKir6.2 is a dominant-negative mutation in regulating K_ATP_ channel trafficking towards plasma membrane.

### Functional characterization of hKir6.2 (A28V) in the *Xenopus* oocytes

**Figure 4.**
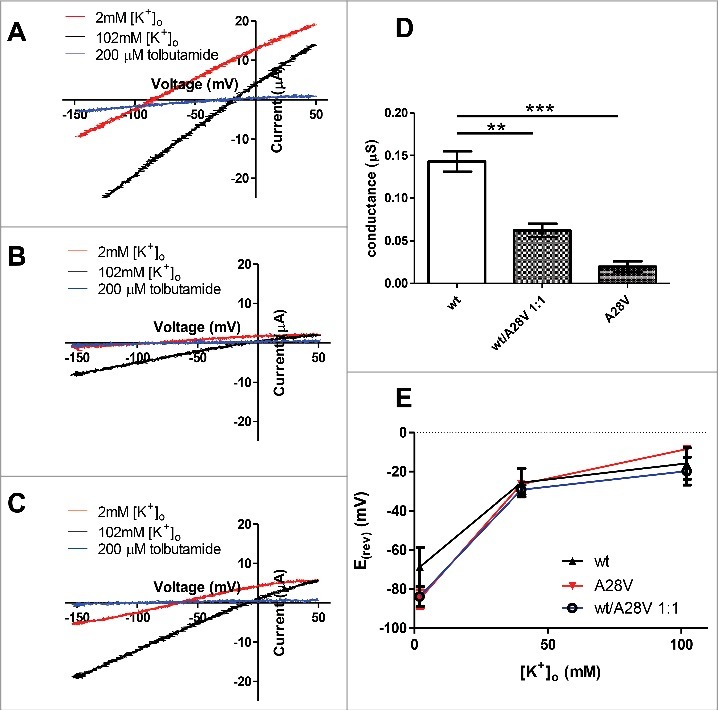
Two-electrode voltage clamp recording of K_ATP_ currents in the Xenopus oocytes. (A) A representative wild-type K_ATP_ current (red trace) was elicited by a voltage ramp pulse (0.5V/s). As predicted, the K_ATP_ current was inhibited by 200 µM K_ATP_ channel blocker, tolbutamide (blue trace). Shifting extracellular potassium concentration from 2 mM to 100 mM caused a shift of reversal potential, as predicted by the potassium equilibrium potential. (B) A representative A28V hKir6.2 K_ATP_ current (red trace) was elicited by a voltage ramp pulse (0.5V/s). This A28V hKir6.2 K_ATP_ current was relatively smaller than the wild-type K_ATP_ current, but it could be inhibited by 200 µM tolbutamide (blue trace). The K_ATP_ channels containing A28V hKir6.2 were still potassium selective, as the reversal potential followed the potassium equilibrium potential (black trace). (C) A representative recording trace from a Xenopus oocyte injected with an equimolar ratio of wt and A28V hKir6.2 mRNA. K_ATP_ current (red trace) was elicited by a voltage ramp pulse (0.5V/s). This wt/A28V K_ATP_ current size was in between wild-type and A28V hKir6.2 K_ATP_ current, and was inhibited by 200 µM tolbutamide (blue trace). The wt/A28V K_ATP_ currents were also potassium selective, as the reversal potential followed the potassium equilibrium potential (black trace). (D) Summary of wt, A28V and wt/A28V K_ATP_ currents in the Xenopus oocytes. wt K_ATP_ currents (n = 7) were significantly larger than A28V K_ATP_ currents (n = 6) and wt/A28V K_ATP_ currents (n = 8), as determined by one-way ANOVA with posthoc multiple comparison test, (**p = 0.001, ***p < 0.0005). (E) Summary of the reversal potentials of wt, A28V, and wt/A28V K_ATP_ currents. In all three groups, the reversal potentials followed closely with the extracellular potassium concentration, indicating this hKir6.2(A28V) mutant is still potassium selective.

Since the expression of A28V hKir6.2 in HEK cells generated minuscule currents that were too small to characterize the biophysical and pharmacological properties of the mutant channels, we sought to express A28V-hKir6.2 in another cell type that normally shows more permissive membrane trafficking regulation. At lower temperature, the *Xenopus* oocyte exhibits less stringent trafficking control on membrane protein.^14,15^ allowing for recording of K_ATP_ currents formed by the A28V-hKir6.2 in the *Xenopus* oocytes. Indeed, when equal mass amounts of RNA were injected into the *Xenopus* oocytes, the A28V-hKir6.2 containing K_ATP_ currents were detectable but significantly smaller than the wild-type K_ATP_ currents (Fig. 4A and C). We found that the K_ATP_ currents formed by A28V hKir6.2 can be blocked by a specific K_ATP_ channel inhibitor, tolbutamide, indicating pharmacological properties are still well-preserved in these mutant channels when trafficked to the plasma membrane (Fig. 4B and D).

Another plausible mechanism that could contribute to the etiology of PHHI would be if potassium selectivity is impaired in A28V hKir6.2 containing channels. Under this scenario, mutant channels that lose potassium selectivity could cause depolarization rather than hyperpolarization.^16^ To characterize the ion selectivity in K_ATP_ channels, we injected hSUR1, together with either wild-type or A28V hKir6.2, into the *Xenopus* oocytes and measured the K_ATP_ currents in the extracellular ND96 solutions with different potassium concentrations. We found that the reversal potentials of the wild-type and A28V hKir6.2 containing K_ATP_ currents closely followed the potassium equilibrium potentials (Fig. 4A, B and E), indicating that the K_ATP_ channels formed by A28V hKir6.2 channels remain highly selective for potassium. Finally, we tested whether co-injecting a 1:1 ratio of wild-type and A28V-hKir6.2 would reduce K_ATP_ currents in the *Xenopus* oocytes. Indeed, we observed reduced K_ATP_ currents in the oocytes that were co-injected with wild-type and A28V-hKir6.2 RNAs, which further supported the hypothesis that A28V-hKir6.2 is a dominant-negative mutation (Fig. 4B).

### A28V suppresses hKir6.2 surface expression via reorienting the ER-retention motif

**Figure 5.**
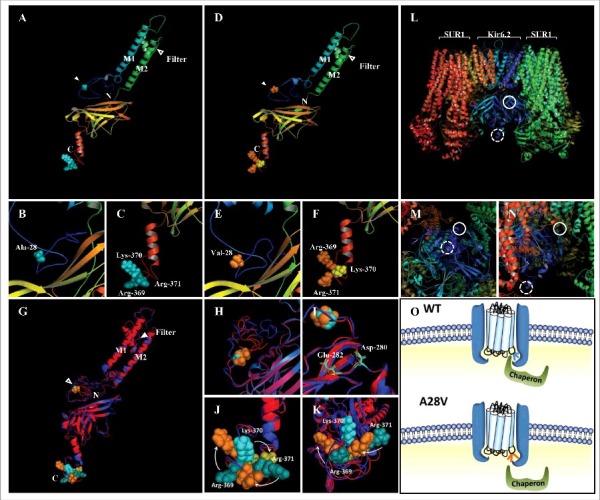
Predicted protein structures of wild-type (A to C) and A28V (D to F) hKir6.2. The first and second transmembrane segments are labeled as M1 and M2, respectively. The selective filter and the position 28 are marked with blank and filled arrowhead, respectively. The N and C represent the N- and C- terminus of the hKir6.2 protein (A and D). (G to K) A superimposed image of the predicted wild-type and A28V hKir6.2. The side chain of position 28 (alanine in wild-type and valine in the mutant) are shown as cyan balls for wild-type and orange balls for the mutant (H and I). Di-acidic motifs of wild-type and A28V hKir6.2 (D280 and E282) are shown as cyan and orange sticks, respectively. (J) The A28V mutation caused a clockwise rearrangement of the RKR motif. (L to N) The fully assembled K_ATP_ channel complex (PDB: 5WUA).18 The solid and dashed circles represent the presumptive N-terminal region containing the 28th alanine residue and C- terminal region containing the RKR motif, respectively. (O) A plausible molecular mechanism of the A28V mutation on K_ATP_ channel trafficking. In K_ATP_ channel formed by the wild-type Kir6.2, the RTR motif is hinged on the neighboring SUR1, and the chaperone may dock onto the K_ATP_ channel complex to facilitate the assembled channel complex exiting the ER. In K_ATP_ channel formed by the A28V Kir6.2, the C-terminus is distorted and the RKR motif is no longer hidden. The exposed RKR motif may cause a hindrance for the chaperone docking and hence, prevent the forward trafficking of the mutated K_ATP_ channel.

Since the A28V hKir6.2 mutation dramatically decreased its surface expression, we wondered whether this mutation might alter trafficking signals such as the arginine-lysine-arginine (RKR) ER-retention motif^12^ or the di-acidic ER-exit motif.^17^ Although the high-resolution structure of the K_ATP_ channel has been solved recently,^18,19^ the detailed structure of N- and C-terminal regions are yet to be determined. We utilized *de novo* protein structure prediction method^20–23^ to construct the N- and C-terminal regions of hKir6.2 according to the existing atomic K_ATP_ channel structures (Fig. 5).^18^ As illustrated in Fig. 5, our model predicted that the N-terminal loop (solid circle) and the C-terminal loop (dashed circle) of the hKir6.2 were surrounded by the ATP-binding pocket at the hKir6.2 and the inter-subunit interface between hKir6.2 and SUR1 (Fig. 5L–N). Alanine to valine mutation at position 28 had little effect on the overall Kir6.2 structure (Fig. 5A–F). However, a superimposition of the wild-type and A28V hKir6.2 structures (Fig. 5G) revealed that the A28V mutation caused a pronounced conformational change at the N-terminal loop (Fig. 5B, E and H) and a counterclockwise rotation and outward shift of the RKR ER-retention motif (Fig. 5 C, F, J and K). On the contrary, A28V mutation only produced a minute change on the ER-exit di-acidic motif (Fig. 5I).

## Discussion

In this study, we have identified the first dominant-negative mutation in *KCNJ11*, the gene that encodes the pore-forming subunit of the K_ATP_ channels, hKir6.2. Patients that carry one copy of this *KCNJ11*(C83T) mutation exhibited various symptoms, ranging from a mild PHHI to hypothalamic deficiency.

The atomic structures of the fully assembled K_ATP_ channel in the closed conformation provide detailed structural and mechanistic insights into K_ATP_ channel assembly and gating.^18,19^ Nevertheless, the regions that are involved in K_ATP_ channel trafficking have yet to be determined, as these trafficking domains are highly dynamic structures. The predicted full-length hKir6.2 structure indicated that the A28 is located on the loosely-packed N-terminal loop; Ala to Val not only re-organizes the N-terminal loop but also distortes the entire C-terminal domain (Fig. 5J and K). We postulate that the distorted C-terminal domain hinders the docking of the chaperones to the RKR ER-retention motif and consequently prevents the K_ATP_ channels trafficking to the plasma membrane (Fig. 5O). The trafficking defect may also explain the dominant-negative effect of the A28V mutation, since the forward trafficking of the fully assembled K_ATP_ channels can only take place when all the RKR ER-retention motifs on the Kir6.2 and SUR1 are masked.^12,24^ Exposure of any RKR ER-retention motif traps the channel complex in the ER and reroutes the defected channel to the degradation pathway instead.^25^

Multiple mutations have been identified in both genes encoding K_ATP_ channel subunits (i.e., *KCNJ11* and *ABCC8*) with many of these strongly affect glucose homeostasis.^26^ Mutations in *KCNJ11* can lead to either hyper- or hypo-insulin secretion, depending on their nature.^2,27,28^ However, mutations in *ABCC8* mostly result in hyperinsulinemia.^2,3,11^ Kir6.2 has evolved to be a high-affinity receptor for [ATP]_i_.and Kir6.2 mutations located near the ATP binding pocket often render the K_ATP_ channel less sensitive to [ATP]_i_.^29–30^ As the closure of the K_ATP_ channels is a critical link in the pathway by which glucose stimulates insulin secretion, ATP-insensitive K_ATP_ channels that remain open keep pancreatic β-cells hyperpolarized even at elevated blood glucose levels. Consequently, glucose fails to stimulate insulin secretion, leading to the development of diabetes.^31^

Conversely, inactivating mutations in the Kir6.2 gene often cause PHHI, since β-cells without functional K_ATP_ channels are depolarized, and continue to secrete insulin at low glucose concentration.^3^ In contrast, most SUR1 mutations lead to PHHI.^3,11,26,32^ SUR1 is a high-affinity receptor for intracellular magnesium nucleotides, such as Mg-ATP and Mg-ADP. Binding of the magnesium nucleotides to the SUR1 holds the K_ATP_ channels in the open state, even in the presence of physiological [ATP]_i_ that are up to an order of magnitude higher than the normal IC_50_ of [ATP]_i_.^33^ In addition, many mutations in genes encoding Kir6.2 and SUR1 are recessive, thus requiring inheritance of two mutated alleles to exhibit clinical symptoms or signs of disease. The newly identified A28V hKir6.2 mutation is a striking exception to often-observed paradigms in K_ATP_ channelopathy: (1) A28V hKir6.2 is a maternally-inherited dominant negative mutation. The index patient and his immediate family members who carry one copy of the same A28V mutation all developed hypoglycemia. (2) K_ATP_ channels containing A28V hKir6.2 remain functional if the channel reaches the cell membrane (Fig 4).

In addition to persistent low blood glucose, the index patient and his sister also exhibited some degree of pituitary insufficiency may be secondary to hypothalamic effects. Kir6.2 is highly expressed in many excitable tissues, including pancreas, brain, muscle and heart.^34^ The K_ATP_ channels in neurons and pancreatic β-cells are similar, in that they are formed by Kir6.2 and SUR1. In cardiac muscle, K_ATP_ channels are formed by Kir6.2 and SUR2A.^7^ The differential expression of SUR subunits may also contribute to breadth of pathologies induced by K_ATP_ channelopathy. Since Kir6.2 is expressed in the hypothalamus,^35,36^ we expect the expression of the dominant negative A28V hKir6.2 protein in hypothalamic neurons could impact the K_ATP_ channels, disrupt hypothalamic function and thereby effect pituitary function. Previous studies have identified multiple roles for K_ATP_ channels in the hypothalamus, ranging from regulation of food intake and glucose homeostasis to hormonal functions.^36,37^ As the hypothalamus is heavily involved in regulating peripheral glucose homeostasis,^38^ we also cannot rule out the potential involvement of the hypothalamus in PHHI.^39^ A similar argument could be made for secretion from the enteroendocrine cells that might explain the gastrointestinal pathology in the index patient.

Most PHHI patients develop clinically evident hypoglycemia within a few days of birth. By contrast, the index patient for the A28V hKir6.2 mutation presented with non-specific symptoms at 9 months and was not diagnosed with PHHI until more than one year after birth, and his older sibling not diagnosed until 12 months of life. One potential explanation for the delayed onset is that the A28V hKir6.2 mutation affects K_ATP_ channel trafficking, and the molecular machinery for proper channel trafficking might not be fully developed in infants. In an animal study, mouse β-cells did not exhibit adult-like membrane excitability until postnatal day P3.^40^

This patient and his affected sister did not respond to the K_ATP_ channel opener diazoxide which is often prescribed to manage PHHI symptoms. This failure of diazoxide can be explained by our results. The K_ATP_ channels carrying A28V hKir6.2 still had normal pharmacological responses, but the channels did not correctly traffic to the cell surface. This lack of functional K_ATP_ channels on the β-cell surface removes the target of activity modulating drugs and makes the affected patients refractory to PHHI medications.

This A28V hKir6.2 mutation affects channel trafficking, and until now, only limited number of therapeutic strategies have been developed to alleviate protein trafficking defects. Lumacaftor, a newly developed small molecule drug and the first medicine to correct CFTR trafficking defects, has been successfully integrated into clinical practice for the treatment of patients with specific CFTR mutations (DelF508).^41^ Lumacaftor functions as a molecular chaperone and forces the defective channels to travel to the membrane. While it represents a promising start, this drug only benefits ∼4% of CFTR patients that carry this specific CFTR mutation.^42^ *In vitro* studies have demonstrated that carbamazepine, an antiepileptic drug, and sulphonylureas such as tolbutamide and glibenclamide may function as molecular chaperones to correct certain K_ATP_ channel trafficking defects.^43–46^ We predict that those molecular chaperones that promote forward trafficking may be beneficial for patients with K_ATP_ channels that exhibit trafficking defects.

In summary, we have identified a novel form of late-onset, dominant-negative A28V hKir6.2 mutant that impairs K_ATP_ channel trafficking. Patients carrying this mutation developed a novel form of PHHI and hypopituitarism. With the rapid improvement of personalized medicine, we expect novel therapeutic strategies can be developed to treat patients with this K_ATP_ channel trafficking defect.

## Materials and methods

### Whole-cell patch clamp recording in HEK cells

Human Kir6.2 (hKir6.2), SUR1 (hSUR1) (Origene, USA) and rodent (hamster) SUR1 (rSUR1) cDNAs were cloned into the pcDNA3 plasmid. The HA-tagged rSUR1 was generated previously and was kindly provided as a gift from Dr. Lily Jan at the University of California, San Francisco, USA. The HA-epitope was inserted between the sixteenth and seventeenth transmembrane domains of the SUR1 subunit and the sequence of the HA-tag reads as^1272^LHRELSAGLV**YPYDVPDYAHRELSAGLV**GLG^1284^at the site of the epitope insertion.^12^ Site-directed mutagenesis was performed using Pfu Turbo DNA polymerase (Stratagene, USA) and the A28V mutation was verified by sequencing. HEK293 cells were cultured in DMEM (Thermo Fisher Scientific, USA) containing 10% FBS (Hyclone, USA), 2 mM glutamine, 100 units penicillin and 100 mg/ml streptomycin in a humidified atmosphere of 5% CO_2_ at 37°C. Cells were plated on poly L-ornithine-coated glass coverslips and transiently transfected with 0.2 μg of the pcDNA3 containing hKir6.2 construct and 0.8 μg of pcDNA3 containing SUR1 construct by using FuGENE 6 (Roche, USA). Cells were used 2–4 days after transfection. K_ATP_ currents were recorded using the whole-cell patch-clamp configuration by an Axopatch 700B amplifier (Molecular Device, USA) in a standard extracellular solution containing: 150 mM NaCl, 10 mM HEPES, 5 mM KCl, 2 mM CaCl_2_, 1 mM MgCl_2_ and pH 7.2, adjusted with NaOH.^47^ Data were acquired at 10 kHz with pCLAMP software (Molecular Device, USA). Pipettes were pulled from 1.5 mm borosilicate glass capillaries (Sutter Inc, USA). Pipette resistances were 2–4 MΩ when filled with the intracellular solution containing: 135 mM K gluconate, 15 mM KCl, 10 mM HEPES, 5 mM Mg_2_ATP, 1 mM Na_3_GTP, 10 mM sodium phosphocreatine, 0.05 mM EGTA and pH 7.2, adjusted with KOH. The access resistances of whole-cell recording ranged between 5 and 20 MΩ and were compensated by ∼80%. All experiments were performed at room temperature (∼25°C). For all studies, all chemicals were purchased from Sigma-Aldrich (USA) if not stated otherwise.

### Two electrode recording in Xenopus oocytes

Animal protocols used in this study were approved by the IACUC at Academia Sinica or University of California, San Francisco, and Xenopus oocytes were prepared as described previously.^47^ Briefly, stage V–VI *Xenopus laevis* oocytes were harvested, injected with 30 ng of each cRNA, and incubated at 16°C for 2–4 days before recording. hKir6.2 and hSUR1 were cloned into the pGEM vector. hKir6.2 and hSUR1 cDNAs were first linearized by restriction enzyme digestion, and cRNA was then synthesized by using the mMESSAGE mMACHINE SP6 Transcription kit (Thermo-Fisher, USA). Macroscopic currents were recorded from oocytes with two-electrode voltage clamp (GeneClamp 500B, Molecular Device, USA). Electrodes were filled with 3 M KCl and had a resistance between 0.4–1 MΩ. A small chamber with a fast perfusion system (AutoMate Scientific, USA) was used to change extracellular ND96 solution containing: 96 mM NaCl, 2 mM KCl, 1 mM MgCl_2_, 5 mM HEPES and pH 7.4, adjusted with NaOH (in some cases, the Na^+^ was replaced with equimolar K^+^ to keep the osmolarity constant).

### Immunofluorescent staining

For non-permeabilized immunostaining of surface K_ATP_ channels, HEK cells were first transfected with hKir6.2 and an HA-tagged rSUR1. Three days post transfection, cells were incubated in the blocking solution (5% normal goat serum in PBS; Jackson ImmunoResearch Laboratories, INC. USA) for 10 min at 4°C and then incubated with rat anti-HA antibody (1:200; monoclonal 3F10, Sigma-Aldrich, USA) in the blocking solution for 30 min at 4°C to minimize endocytosis. After washing four times with PBS for 5 min each at 4°C, cells were incubated for 30 min with goat anti-rat Alexa488 secondary antibody (1:1000, Invitrogen, USA) then washed three times with PBS for 5 min each at 4°C. Next, the cells were fixed in the 4% paraformaldehyde/4% sucrose in PBS for 10 min at room temperature then washed three times with PBS for 5 min each. Fixed cells were permeabilized by incubating in blocking solution that contained 0.1% Triton-X-100 for 15 min. Then cells were incubated with the same rat anti-HA antibody (1:200) in the blocking solution for 30 min at room temperature. After washing four times with PBS for 5 min each at room temperature, cells were incubated for 30 min with goat anti-rat Alexa555 secondary antibody (1:1000 Invitrogen, USA), then washed three times with PBS for 5 min each at room temperature. The coverslips were mounted using Fluoromount G mounting medium containing DAPI (Southern Biotech, USA) and images were acquired using a confocal microscope (Zeiss, Germany).

### *De novo* protein structure prediction

The structures of the wild-type and A28V hKir6.2 were predicted by I-TASSER^21–23^ and the rat Kir6.2 (PDB: 5WUA)^18^ was used as a template for *de novo* structure prediction. TM-Align was used to align wild-type and A28V hKir6.2.^20^ Predicted structures were generated in Pymol.

### Data analysis

Imaging data from the immunostained cells were analyzed using ZEN software (Zeiss, Germany). Electrophysiology Data were analyzed with pClamp10 software (Molecular Devices Corp., USA). Results are reported as mean ± SEM. Statistical analysis was performed using Prism 5 (GraphPad, USA), with differences considered significant at *p* < 0.05 (*p < 0.05, **p < 0.01, ***p < 0.001 in all graphs).
